# Using interpretable survival analysis to assess hospital length of stay

**DOI:** 10.1186/s12913-025-12852-0

**Published:** 2025-05-22

**Authors:** Yan Li, Trevor Hall, Fahad Razak, Amol Verma, Mark Chignell, Lu Wang

**Affiliations:** 1 Department of Mechanical and Industrial Engineering, University of Toronto, Toronto, ON Canada; 2 Healthcare Insurance Reciprocal of Canada, Toronto, ON Canada; 3 The General Medicine Inpatient Initiative (GEMINI), Unity Health Toronto, Toronto, ON Canada; 4 Institute of Health Policy, Management and Evaluation, University of Toronto, Toronto, ON Canada; 5 Department of Biomedical Engineering, University of Houston, Houston, TX USA; 6 Department of Health Systems & Population Health Sciences, University of Houston, Houston, TX USA

**Keywords:** Interpretable machine learning, Survival analysis, Hospital length of stay

## Abstract

Accurate in-hospital length of stay prediction is a vital quality metric for hospital leaders and health policy decision-makers. It assists with decision-making and informs hospital operations involving factors such as patient flow, elective cases, and human resources allocation, while also informing quality of care and risk considerations. The aim of the research reported in this paper is to use survival analysis to model General Internal Medicine (GIM) length of stay, and to use Shapley value to support interpretation of the resulting model. Survival analysis aims to predict the time until a specific event occurs. In our study, we predict the duration from patient admission to discharge to home, i.e., in-hospital length of stay. In addition to discussing the modeling results, we also talk about how survival analysis of hospital length of stay can be used to guide improvements in the efficiency of hospital operations and support the development of quality metrics.

## Introduction

Analyzing data from large healthcare repositories may offer valuable insights into the sources of inefficiencies and inequities within healthcare systems. One of the key challenges in healthcare is the diverse ways in which quality can be measured. While cost, in terms of dollars, is a relatively accessible indicator of efficiency, it can vary significantly due to factors like geographic location, hospital pricing policies, and payer agreements. On the other hand, in-hospital length of stay (LOS)-the duration between a patient’s admission and discharge-is a straightforward metric that can be uniformly applied across different healthcare systems to assess the quality and effectiveness of healthcare services. Analyzing LOS across different demographic and socioeconomic groups can also reveal disparities in quality of care and access to services, ultimately contributing to improved health equity.

**Fig. 1:**
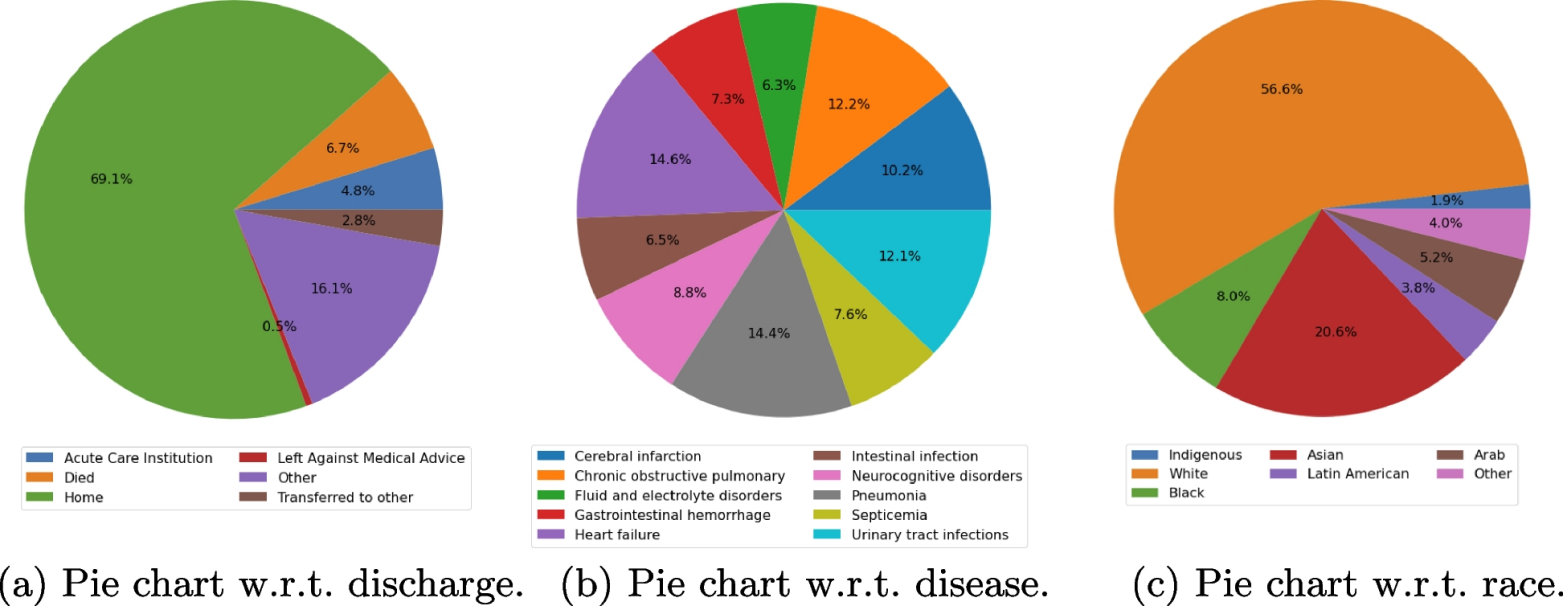
The distribution of GEMINI data with regard to (w.r.t.) the discharge type (**a**), disease (**b**), and race (**c**) respectively

Predicting LOS can be formulated as a survival analysis problem, which aims to predict the time to a specific event-of-interest [1]. In our study, the event-of-interest is discharge to home. The dataset used in the research is the General Medicine Inpatient Initiative (GEMINI) data repository. The GEMINI repository contains detailed clinical and administrative data from more than 30 participating hospitals across Ontario, Canada. The GEMINI data that we used covered six discharge categories: home, death, acute care institution, transfer to another facility, discharge against medical advice, and other, as shown in Fig. 1a. We chose discharge to home as the event-of-interest because it indicates that a patient’s condition has sufficiently improved, or been resolved, to the point that inpatient care is no longer needed. Other discharge types are inappropriate for assessing quality and effectiveness of healthcare services because they are less successful outcomes which may reflect increased frailty or death after treatment (e.g., death, acute care institution, transfer to another facility, left against medical advice), or loss of tracking (e.g. other). Thus patients who died in the hospital or were discharged under any of the other four discharge types were considered as censored instances.

Because of the presence of *censoring*, conventional statistical and machine learning predictive models are not directly applicable for analyzing survival data. Survival analysis enhances conventional regression models by taking into account both instances where target events are observed (uncensored instances) and instances that are not observed (censored instances). This approach utilizes more information than standard regression models, leading to more robust predictions.

In this research, we focus on predicting the LOS for patients with conditions commonly addressed in the General Internal Medicine (GIM) specialty. Specifically, we examine ten frequently observed diseases that are covered by the discipline of internal medicine. The ten diseases examined were: cerebral infarction, chronic obstructive pulmonary disease and bronchiectasis, fluid and electrolyte disorders, gastrointestinal hemorrhage, heart failure, intestinal infection, neurocognitive disorders, pneumonia, septicemia, and urinary tract infections, with the proportions of each disease type appearing in the data set used in our analyses shown in the middle panel of Fig. 1a. After showing how survival analysis can be used to predict LOS, we will then discuss how the interpretation method can be used to improve the model’s explainability by identifying the most important predictors for the LOS prediction. Model explainability is important to healthcare providers because it provides interpretability, and if the interpretation is consistent with expectations and background knowledge, then the model will be seen to be more trustworthy and there will be great confidence in the predicted results.

In our study, we began with a descriptive analysis using Kaplan-Meier curves [2] to examine the survival curves for each disease in the dataset. This analysis allowed us to assess the variability in-hospital LOS across different diseases. The results of this analysis provided insights into whether 1) a single model for all diseases was adequate (if variability was minimal) or 2) tailored models for each disease were required (if variability was substantial). We then implemented five methods to predict LOS for each patient: the Standard Cox model [3], the XGBoost-enhanced Cox model [4], the Random Survival Forest (RSF) [5], DeepSurv [6], and CoxTime [7]. The Standard Cox proportional hazards model was included as it is a standard method for survival analysis. The XGBoost-enhanced Cox model and RSF are tree-based ensemble methods known for their ability to model non-linearities and generally perform well with tabular data [8]. DeepSurv and CoxTime are newer deep learning models that extend the Cox model to better handle non-linear relationships between input features and the outcome variable. The concordance index (C-index) was used to evaluate the performance of the survival analysis models [9]. Finally, the Shapley Additive Explanations (SHAP) [10] method was employed to interpret the best performing model (which was the XGBoost-enhanced Cox model in our analysis) in terms of feature importance.

The research reported in this paper shows how survival analysis can support hospital leaders and health policy decision-makers with modeling LOS. Besides LOS prediction, survival analysis has been widely applied to various healthcare problems, including modeling disease progression, identifying prognostic factors, and assessing health risks. In the discussion section, we will also explore how survival analysis can guide projects aimed at improving hospital efficiency or identifying health equity concerns.

## Methods

This section introduces the fundamental concepts of survival analysis, followed by a brief discussion of the methods used in this paper.

### Overview and notations

In survival analysis, for each data instance (such as a patient), we can observe either a *survival time*$(O_i)$ or a *censored time*$(C_i)$. In non-recurrent event survival analysis, an instance is typically represented as a triplet $(X_i, T_i, \delta _i)$, where $X_i \in \mathbb {R}^{1 \times p}$ denotes the feature vector and $\delta _i$ is the censoring indicator, which equals 1 for an uncensored instance and 0 for a censored one. The dataset is right-censored if and only if the *observed time*$T_i = \min (O_i, C_i)$ is recorded during the study.

The main objective of survival analysis is to develop a predictive function $f_{\Theta }(\cdot )$ parameterized by $\Theta$, such that the predicted survival time closely aligns with the actual survival time. To achieve this, the learning process involves estimating the parameter $\Theta$ by minimizing the empirical expectation of a loss function $\mathcal {L}(\Theta; X,T, \delta )=\sum _{i=1}^N \ell _{sur} (f_\Theta (X_i), T_i, \delta _i))$, where *N* is the number of training instances. The loss function $\ell _{sur}(\cdot )$ is specifically designed for survival analysis, utilizing both uncensored instances (where true survival times are known) and censored instances (where true survival times are unknown but must exceed the corresponding censored times).

The survival function and hazard function are the two primary functions used in survival analysis. The survival function represents the probability that the time to the event of interest is at least *t*, and is formally defined as $S(t)=Pr(O\ge t)$. It is a monotonic non-increasing function, as can be seen in the Kaplan-Meier curve presented in the results section. The hazard function, on the other hand, represents the rate at which events occur at time *t*, given that no event has happened before that time. It is formally defined as:

1 $$\begin{aligned} h(t)= \underset{\Delta t \rightarrow 0}{\textrm{lim}}\frac{Pr(t\le O \le t+\Delta t| O \ge t)}{\Delta t}. \end{aligned}$$

### Kaplan-Meier method

The Kaplan-Meier (KM) method is the most widely used nonparametric method for estimating survival functions [2]. Let $O_1<O_2<\cdots <O_K (K\le N)$ represent a set of distinct ordered survival times for $N (K \le N)$ instances. This ordered list indicates the moments at which events of interest occur among the *N* instances. In addition to these ordered survival times, there are also censoring times for instances where the events of interest have not been observed. At a specific time $O_j~(j=1,2,\cdots,K)$, $d_j\ge 1$ denotes the number of observed events of interest, and $r_j$ represents number of instances “at risk”, including those whose event or censoring times are greater than or equal to $O_j$. It is important to note that, due to the presence of censoring, $r_j \ne r_{j-1}-d_{j-1}$. Instead the correct calculation is $r_j=r_{j-1}-d_{j-1}-c_{j-1}$, where $c_{j-1}$ is the number of censored cases between $O_{j-1}$ and $O_{j}$. The conditional probability of surviving at time $O_j$ can then be defined as:

2 $$\begin{aligned} p(O_j)=\frac{r_j-d_j}{r_j}. \end{aligned}$$

Based on this conditional probability, the product-limit estimate of survival function $S(t)=P(O \ge t)$ can be expressed as follows:

3 $$\begin{aligned} \hat{S}(t)=\prod _{j:O_j<t}p(O_j)=\prod _{j:O_j<t}(1-\frac{d_j}{r_j}). \end{aligned}$$

Alternatively, this can be represented using a recursive formula:

4 $$\begin{aligned} \hat{S}(t_j)=\hat{S}(t_{j-1})(1-\frac{d_j}{r_j}), \end{aligned}$$

where $\hat{S}(t_j)$ and $\hat{S}(t_{j-1})$ are the survival probabilities at time $t_j$ and $t_{j-1}$, respectively.

### Survival prediction methods

#### The standard Cox model

The Cox proportional hazards model is a widely used semi-parametric method for survival prediction [3]. For instance *i*, represented as $(X_i,y_i,\delta _i)$, the hazard function $h(t,X_i)$ in the Cox model is formulated as:

5 $$\begin{aligned} h(t,X_i)=h_0(t)exp(X_i\beta ), \end{aligned}$$

where $h_0(t)$ is the *baseline hazard function* which can be an arbitrary non-negative function of time. $X_i \in \mathbb {R}^p$ is the feature vector, and $\beta ^T \in \mathbb {R}^p$ is the coefficient vector. The Cox model is a semi-parametric model since the baseline hazard $h_0(t)$ is unspecified. For two instances $X_1$ and $X_2$, the hazard ratio is

6 $$\begin{aligned} \frac{h(t,X_1)}{h(t,X_2)}=\frac{h_0(t)exp(X_1\beta )}{h_0(t)exp(X_2\beta )}=\frac{exp(X_1\beta )}{exp(X_2\beta )}=exp[(X_1-X_2)\beta ], \end{aligned}$$

which is independent of the baseline hazard function. Since the hazard ratio is time-invariant and all subjects share the same baseline hazard, the Cox model is a proportional hazards model.

Since the baseline hazard function $h_0(t)$ is unspecified, the coefficient $\beta$ vector cannot be fitted using the standard likelihood function. To estimate $\beta$, a partial likelihood estimator [3, 11] is applied. Let $X_j$ be the feature vector for the *j*-th instance whose event of interest occurs at time $O_j$, and let $\mathcal {R}_j$ be the set of instances “at risk” at that time. The hazard ratio between the *j*-th instance and all “at risk” instances is formulated as:

7 $$\begin{aligned} \frac{h(O_j,X_j)}{\sum _{i\in \mathcal {R}_j}h(O_j,X_i)}=\frac{h_0(O_j)exp(X_j\beta )}{\sum _{i\in \mathcal {R}_j}h_0(O_j)exp(X_i\beta )}=\frac{exp(X_j\beta )}{\sum _{i\in \mathcal {R}_j}exp(X_i\beta )}. \end{aligned}$$

Given the fact that the event or censoring times of all “at risk” instances are greater than $O_j$, the above hazard ratio should be maximized. This maximization indicates that the *j*-th instance has a relatively higher risk of the event occurring compared to the risk among the instances that are still at risk. Considering all *N* instances the partial likelihood is defined as:

8 $$\begin{aligned} L(\beta )=\prod _{j=1}^N\left[ \frac{exp(X_j\beta )}{\sum _{i\in \mathcal {R}_j}exp(X_i\beta )}\right] ^{\delta _j}. \end{aligned}$$

It should be noted that if $\delta _j=1$, the *j*-th term in the product is the hazard ratio, when $\delta _j=0$, the corresponding term is 1 and has no effect on the result. The coefficient vector $\hat{\beta }$ is estimated by maximizing the partial likelihood, or more efficiently, by minimizing the *log-partial likelihood*

9 $$\begin{aligned} LL(\beta )=-\sum \limits _{j=1}^N\delta _j\cdot \left\{ X_j\beta -log[\sum \limits _{i\in \mathcal {R}_j}exp(X_i\beta )]\right\}. \end{aligned}$$

#### The XGBoost enhanced Cox model

The standard Cox model can only handle linear relationships between features and the targets, which fails to model nonlinear relationships. In this analysis, we employed a scalable and distributed gradient boosting library, i.e., XGBoost [4], to handle nonlinearities and provide more flexibility in survival analysis. More specifically, an ensemble tree based non-parametric model $\hat{f}(X_i)$ was used to model the hazard function, rather than a linear model $X_i\beta$,

10 $$\begin{aligned} h(t,X_i)=h_0(t)exp[\hat{f}(X_i)], \end{aligned}$$

and hence the log-partial likelihood of the XGBoost enhanced Cox model was

11 $$\begin{aligned} LL_{\hat{f}}=-\sum \limits _{j=1}^N\delta _j\cdot \left\{ \hat{f}(X_j)-log[\sum \limits _{i\in \mathcal {R}_j}e^{(\hat{f}(X_i)}]\right\}. \end{aligned}$$

#### DeepSurv

Recent advance in deep learning has led to its widespread applications in many domains including survival analysis. In [6], the DeepSurv model was proposed to enhance standard Cox propotional hazard model by introducing a neural network (i.e., multi-layer perceptron) to model the non-linear relations between features and target. As in the previously mentioned XGBoost enhanced Cox model, the log-partial likelihood of the DeepSurv model can be formulated as Eq. (11); while, in DeepSurv model the $\hat{f}(X_i)$ is a neural network.

#### CoxTime

The above-mentioned standard Cox model and two Cox-based extension models all follow the proportional hazard assumption, i.e., hazard ratio between any two individuals remains constant over time. This assumption simplifies the problem but also provides restrictions. To alleviate this, in [7] the CoxTime model proposed a time-dependent formulation for hazard function:

12 $$\begin{aligned} h(t,X_i)=h_0(t)exp[f(t, X_i)]. \end{aligned}$$

The model in Eq. (12) is no longer a proportional hazards model, while its partial likelihood still follows a similar pattern:

13 $$\begin{aligned} LL_{\hat{f}}=-\sum \limits _{j=1}^N\delta _j\cdot \left\{ \hat{f}(T_j, X_j)-log[\sum \limits _{i\in \mathcal {R}_j}e^{(\hat{f}(T_j, X_i)}]\right\}, \end{aligned}$$

where $T_j$ is the event time of *j*-th instance.

#### Random survival forest

The random survival forest method extended the random forest method by using a forest of survival trees for survival prediction [5]. The final survival estimation is derived from the ensemble cumulative hazard function (CHF) of the out-of-bag data from each tree. For a specific leaf node *l*, the CHF is calculated by the Nelson-Aalen estimator [12]

14 $$\begin{aligned} \hat{H}_{l}(t) = \sum \limits _{t_{i, l}<t}\frac{d_{i, l}}{r_{i,l}}, \end{aligned}$$

where $d_{i, l}$ and $r_{i,l}$ are the number of deaths and individuals at risk at time $t_{i, l}$ in the leaf node *l*, respectively.

### Interpretation method

The Shapley Additive Explanations (SHAP) method is based on game theory, where each feature is treated as a player and the Shapley value measures each player’s contribution to the final outcome [10]. The interpretation of the Shapley value for the *j*-th feature is defined as its contribution $\Phi _j$ to the prediction of this particular instance compared to the average prediction for the dataset. More specifically, let val($\cdot$) be the value function of features, i.e., the likelihood or negative Root-Mean-Square Difference (RMSE), in machine learning models, $\Phi _j$ is calculated as:

15 $$\begin{aligned} \phi _j=\sum \limits _{S\subseteq \{1,...,p\}\setminus \{j\}} \frac{\left| S \right|!(p-\left| S \right| -1)!}{p!}[val(S \cup {j})-val(S)], \end{aligned}$$

where S is a subset of the features used in the model and p is the number of features. We can see that $val(S \cup {j})-val(S)$ assesses the gain resulting from adding the $j^{th}$ feature in S. The order of adding the $j^{th}$ features influences the gain. For example, if S is an empty set then the $j^{th}$ feature may dramatically reduce the RMSE of the prediction, but when S already contains a lot of features then the $j^{th}$ feature may only reduce RMSE slightly. To overcome this issue, the Shapley value is calculated as the average gain for all possible orders. Therefore, it can provide an unbiased estimation of feature importance.

## Dataset, experiments and results

### Dataset

The dataset used in the research was selected from the GEMINI repository. The complete GEMINI data set is accessible at the following link: https://geminimedicine.ca/the-gemini-database/. Note that the GEMINI data is publicly available but restricted to authorized users. Access requires an application, completion of required training, and signing a Data Use Agreement.

We first conducted a brief data exploration to examine the basic characteristics of our selected GEMINI data. As shown in Fig. 1a, 69.1% of patients were discharged with the desired medical outcomes and returned home, 6.7% died during hospitalization, and the remaining 24.2% were either transferred for additional rehabilitation (e.g., to another hospital or acute care facility), discharged against medical advice, or lost to follow-up (labeled as ‘other’). In our study, 69.1% of patients are considered as uncensored instances (those who were successfully discharged to their residences), while the remaining 30.9% were censored instances, as they did not achieve the ideal outcome. The censored ratio for each disease is listed under the “Censored ratio” column in the Table 1. According to an annual report from the Auditor General of Ontario, 74% of inpatients in Ontario are discharged to home [13], and the Canadian Institute for Health Information reports that 5.8% of inpatients across Canada are discharged to acute care institutions [14].

**Table 1:** Descriptions of dataset with respect to each disease

Diseases	No. of samples	Censored ratio
Cerebral infarction	12079	0.536
Chronic obstructive pulmonary	14446	0.219
Fluid & electrolyte disorders	7417	0.226
Gastrointestinal hemorrhage	8703	0.188
Heart failure	17310	0.251
Intestinal infection	7728	0.147
Neurocognitive disorders	10236	0.399
Pneumonia	17138	0.286
Septicemia	8935	0.548
Urinary tract infections	14365	0.28

The dataset that we used contained 118,357 unique admissions (samples), encompassing ten distinct types of diseases within General Internal Medicine: cerebral infarction, chronic obstructive pulmonary disease and bronchiectasis, fluid and electrolyte disorders, gastrointestinal hemorrhage, heart failure, intestinal infections, neurocognitive disorders, pneumonia, septicemia, and urinary tract infections. The number of admissions (samples) for each disease is listed under the “No. of samples” in Table 1, with the corresponding distribution illustrated in Fig. 1b.

**Fig. 2:**
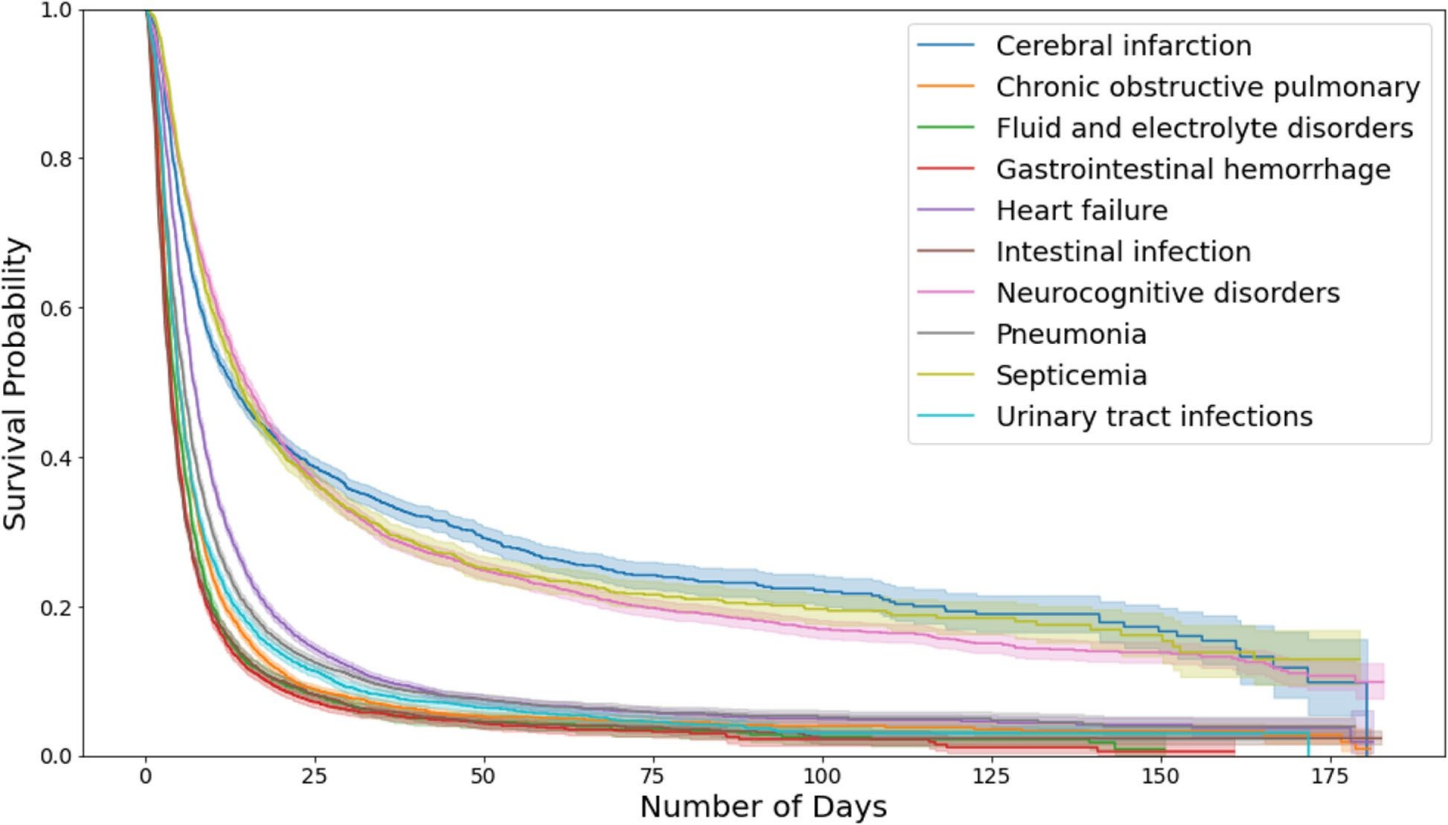
Kaplan-Meier (KM) curves for all patients with different disease types. Patients whose discharge type is not “Home” are considered as censored. Note that as more than 99.5% of patients’ survival time or censored time are less than or equal to half year (183 days), so in this plot the KM curves are truncated at 183 days

In Fig. 2, we show the KM curves of each disease type. Approximately 90% of patients were discharged within 30 days. Patients who suffered from cerebral infarction, neurocognitive disorders, and septicemia stayed in hospital dramatically longer than patients with the other seven types of disease. This reflects the fact that the average in-hospital LOS differs widely between diseases, and hence disease type is a strong predictor for in-hospital LOS.

Since this study focused on analyzing potential LOS during patient admission, we excluded variables that were only accessible during or at the end of the inpatient stay. The preprocessed data included 30 variables from admission data:

1. Ten one-hot-encoded variables were derived from the admission diagnosis disease type, with the corresponding distribution illustrated in Fig. 1b.
2. Nine demographic variables include age (distribution in Fig. 3a), gender (50.83% Female, 49.17% Male), and seven one-hot-encoded race variables: Indigenous, White, Black, Asian, Latin American, Arab, and Other. The racial distribution is shown in Fig. 1c. Since the data were collected from urban hospitals, the race distribution differs from the 2021 Canada census data, which reports 5.0% Indigenous, 69.8% White, 4.2% Black, 14.6% Asian, 1.4% Latin American, 1.8% Arab, and 3.2% Other [15].

**Fig. 3:**
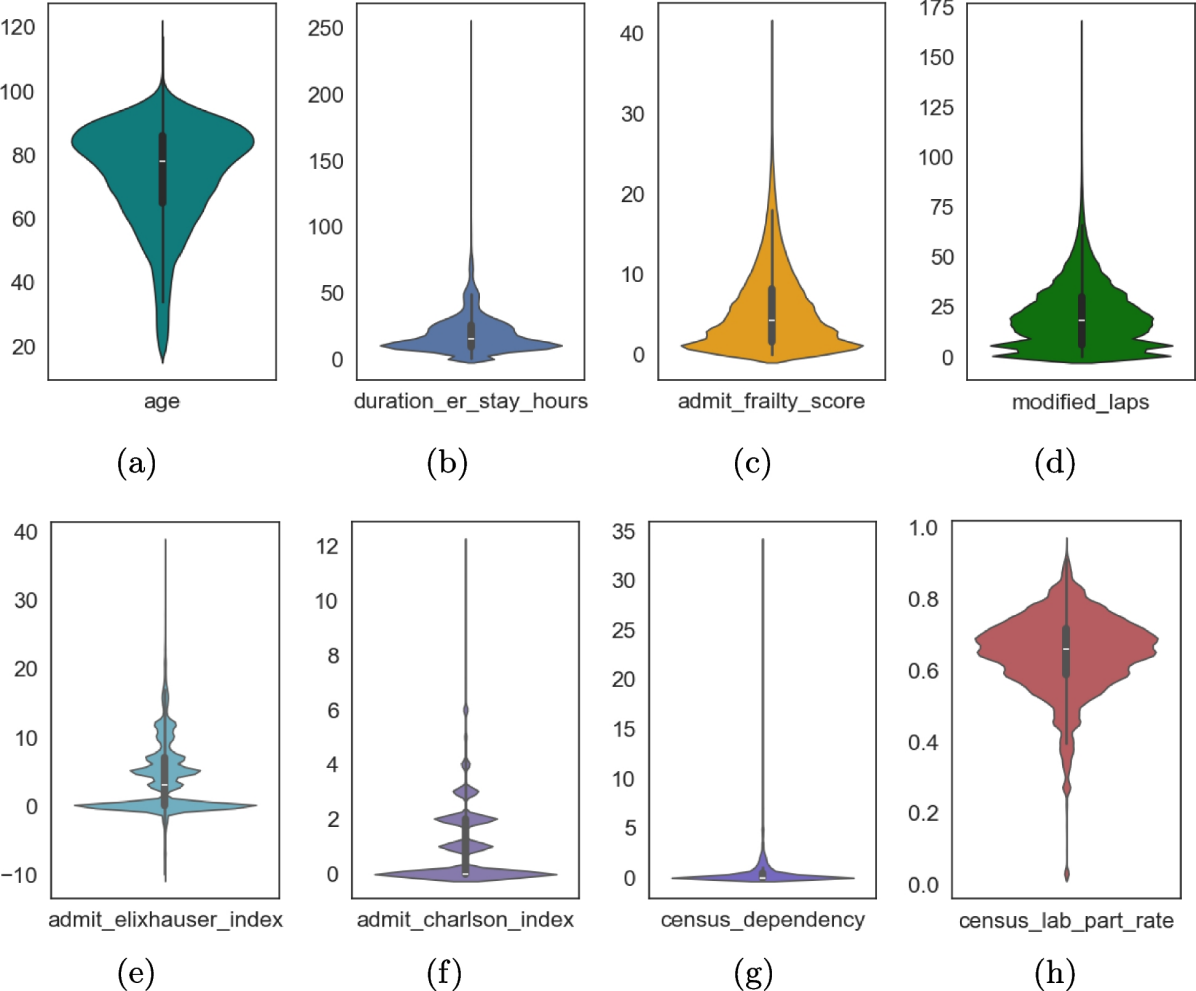
Distribution of eight important non-binary features
3. Five basic vital signs were collected at the time of patient admission: body temperature ($36.87 \pm 0.64 \;^{\circ }C$), heart rate ($68.39 \pm 13.74$ beats per minute (BPM)), systolic blood pressure ($130.05 \pm 23.67$ millimeters of mercury (mmHg)), diastolic blood pressure ($86.83 \pm 16.09$ mmHg), and pain level (ranging from 0 to 10, with an average of $2.08 \pm 2.91$).
4. Two pre-admission variables:
  - “duration_er_stay_hours” stands for the length of stay (in hours) in emergency room, and its distribution in the experimental data is shown in Fig. 3b.
  - “prev_admission_gim_30 d” is the indicator of whether the patient was admitted within 30 days before the current admission. In the dataset, 11.14% of patients had a prior admission within this timeframe.
5. Four commonly used derived healthcare scores and indexes:
  - “admit_frailty_score” is a hospital frailty risk score derived from pre-admission, transfer, and emergency room diagnoses. It screens for frailty and identifies patients at higher risk of adverse outcomes [16]. The distribution in the experimental data is shown in Fig. 3c.
  - “modified_laps” is a modification of the standard Laboratory-based Acute Physiology Score (LAPS). Standard LAPS integrates information from following 14 pre-admission (i.e., emergency department) blood tests: serum albumin, anion gap, arterial pH, PaCO2, PaO2, bicarbonate, total serum bilirubin, blood urea nitrogen, serum creatinine, serum glucose, serum sodium, serum troponin I, hematocrit, and total white blood cell count in thousands [17]. In “modified_laps” the test “serum troponin I” is excluded as it is very sensitive. The distribution of the variable in the experiment is shown in Fig. 3d.
  - “admit_elixhauser_index” is derived from the Elixhauser Comorbidity Index (ECI), calculated using inpatient pre-admission, transfer, and emergency room diagnoses. The ECI categorizes patient comorbidities based on International Classification of Diseases (ICD) codes [18]. A higher score indicates a greater likelihood of mortality or increased resource utilization. In the experimental dataset, the distribution of this variable is shown in Fig. 3e.
  - “admit_charlson_index” is derived from the Charlson Comorbidity Index (CCI), calculated based solely on inpatient pre-admission, transfer, and emergency room diagnoses. Developed in 1987 by Charlson et al., the CCI classifies comorbid conditions that may affect mortality risk [19]. It is the most widely used comorbidity index for assessing survival rates (1-year and 10-year) in patients with multiple comorbidities. The distribution of the “admit_charlson_index” in our experimental data is shown in Fig. 3f.

Additionally, based on the patient’s home address, we linked the admission records with detailed census data to derive 93 variables that reflect the socioeconomic status of the corresponding neighborhood, including factors such as income, education, and employment rates [15]. The distribution of two key socioeconomic variables, “census_dependency” (average number of dependents) and “census_lab_part_rate” (employment rate) in the patient’s residential neighborhood, is shown in Fig. 3g and h, respectively. In summary, after data preprocessing, a total of 123 features were derived.

### Experimental setup

We conducted experiments with the five models listed in “Methods” section. Note that as our target variable (i.e., hospital length of stay) was a continuous variable in the GEMINI dataset, we did not include the discrete-time survival analysis methods such as DeepHit [20], Multi-Task Logistic Regression (MTLR) [21] and MTLSA [22] in our experiments. For each method, the experiments were conducted under two settings: 1) building a disease specific (Local) model for each disease, 2) building a one-size-fits-all (Global) model for all ten diseases. Note that, in all one-size-fits-all models, the disease types are one-hot encoded and the derived features are concatenated to the original feature matrix.

In our experiments, the Standard Cox propotional hazard model was trained using the *coxph* function in the *survival package*^1^ [23]. The XGBoost enhanced Cox Model was implemented in the *XGBoost package*^2^ [4]. The Pytorch implementation of DeepSurv and CoxTime models is available in the *pycox package*^3^ [7]. Note that these two deep learning methods use the same neural network architectures i.e., local models are multi-layer perceptron (MLP) with two 32-node hidden layers and global models are MLP with one 64-node and one 32-node hidden layers, and the dropout rate for all models were 0.2. The implementation of the random survival forest method can be found in the R package *randomForestSRC*^4^ [5]. All data pre-processing and experiments were carried out on a PC equipped with a 2.6 GHz 6-Core Intel i7 CPU, 16GB DDR4 2667 MHz RAM, and 512GB SSD storage, which was found to be sufficient to handle the size of data set (around 120k cases) used in this study.

### Evaluation metric

Because of censoring, conventional regression evaluation metrics like $R^2$ and Mean squared error (MSE) were not well-suited for survival analysis. To deal with this issue, the concordance index (C-index), or *concordance probability*, is commonly used in survival analysis to evaluate the agreement between predictions and observations for pairs of instances [9]. For two instances $(O_1,\,\hat{O}_1)$ and $(O_2,\,\hat{O}_2)$, the concordance probability is defined as:

$$\begin{aligned} c=Pr(\hat{O}_1>\hat{O}_2|O_1 \ge O_2), \end{aligned}$$

where $O_i$ represents the actual survival time, and $\hat{O}_i$ denotes the predicted survival time. In practice, this probability is calculated as the proportion of correctly ordered pairs among all comparable pairs. In the Cox model and its extensions, the hazard ratio is predicted. Instances with lower hazard rates are associated with longer survival; thus, the C-index is calculated as follows:

16 $$\begin{aligned} c=\frac{\sum \limits _{i\in \{1\cdots N|\delta _i=1\}} \sum \limits _{T_j>T_i}I[X_i\hat{\beta }>X_j\hat{\beta }]}{\sum \limits _{i\in \{1\cdots N|\delta _i=1\}} \sum \limits _{T_j>T_i} 1}, \end{aligned}$$

where $I[\cdot ]$ is the indicator function. Note that in the equation, $T_i$ is the survival time of an uncensored instance, while $T_j$ can be either a survival time or a censored time. Thus, in addition to evaluating the concordance of pairs of uncensored instances, the C-index can also assess pairs that include one censored instance with a longer censoring time, making it robust for model evaluation in the presence of censoring.

### Results

Table 2 summarizes the experiment results of four models under the aforementioned two settings, and all the results shown are the average of 10-fold cross validation. As shown in Table 2, the XGBoost enhanced Cox models outperformed the other three models on all 10 diseases. This was an expected result since the XGBoost enhanced Cox model is built based on the gradient boosting method, which is able to handle nonlinearities and hence is more powerful than linear models, and tree ensemble models usually outperform deep learning models in tabular data especially with the existence of categorical features [8]. We can also observe that the disease specific XGBoost enhanced Cox models (XGBoost-Cox Local) outperformed the one-size-fits-all XGBoost enhanced model (XGBoost-Cox Global) on 9 out of the 10 diseases. This indicates that the heterogeneity among these 10 diseases is too great to be handled by indicator variables (i.e., one-hot encoder vectors of disease type) in XGBoost. The average concordance index value across the diseases is around 0.7, which indicates that if we randomly choose a pair of patients the model will give us a correct order of their time to discharge and go back home with 70 percent accuracy. The C-index is a measure of how well a model ranks patients in terms of their risk, with higher values indicating better discriminatory power. An improvement in the C-index means the model can more accurately differentiate between patients at high risk and those at low risk. This enhanced stratification should help healthcare providers prioritize interventions for high-risk patients, potentially reducing adverse outcomes.

**Table 2:** Performance comparison between standard Cox proportional hazard model, XGBoost enhanced Cox model, Deepsurv, CoxTime, and RSF using Harrell’s C-index (along with their corresponding standard deviations)

Diseases	Standard-Cox		Deepsurv		CoxTime		RSF		XGBoost-Cox	
	Local	Global	Local	Global	Local	Global	Local	Global	Local	Global
Cerebral infarction	0.678	0.662	0.694	0.701	0.729	0.713	0.701	0.704	**0.739**	0.714
	(0.031)	(0.031)	(0.035)	(0.034)	(0.039)	(0.037)	(0.044)	(0.046)	**(0.041)**	(0.043)
Chronic obstructive pulmonary	0.645	0.653	0.672	0.669	0.686	0.685	0.667	0.663	**0.707**	0.705
	(0.013)	(0.015)	(0.021)	(0.023)	(0.015)	(0.018)	(0.021)	(0.022)	**(0.025)**	(0.025)
Fluid & electrolyte disorders	0.626	0.622	0.669	0.666	0.669	0.664	0.661	0.663	0.686	**0.696**
	(0.024)	(0.024)	(0.025)	(0.025)	(0.020)	(0.024)	(0.026)	(0.029)	(0.028)	**(0.031)**
Gastrointestinal hemorrhage	0.663	0.651	0.684	0.679	0.687	0.67	0.671	0.669	**0.692**	0.675
	(0.017)	(0.019)	(0.016)	(0.016)	(0.016)	(0.018)	(0.019)	(0.016)	**(0.015)**	(0.016)
Heart failure	0.656	0.654	0.689	0.681	0.688	0.678	0.67	0.651	**0.702**	0.699
	(0.008)	(0.008)	(0.008)	(0.009)	(0.006)	(0.009)	(0.009)	(0.010)	**(0.009)**	(0.009)
Intestinal infection	0.596	0.615	0.646	0.646	0.657	0.66	0.652	0.653	**0.675**	0.646
	(0.018)	(0.020)	(0.018)	(0.021)	(0.019)	(0.019)	(0.018)	(0.016)	**(0.015)**	(0.014)
Neurocognitive disorders	0.645	0.634	0.643	0.653	0.656	0.66	0.643	0.652	**0.685**	0.664
	(0.023)	(0.025)	(0.028)	(0.022)	(0.027)	(0.028)	(0.028)	(0.031)	**(0.031)**	(0.025)
Pneumonia	0.672	0.662	0.676	0.661	0.677	0.675	0.663	0.659	**0.678**	0.675
	(0.023)	(0.029)	(0.025)	(0.027)	(0.025)	(0.030)	(0.025)	(0.026)	**(0.024)**	(0.025)
Septicemia	0.682	0.652	0.702	0.694	0.715	0.694	0.695	0.665	**0.726**	0.709
	(0.027)	(0.027)	(0.027)	(0.027)	(0.027)	(0.029)	(0.027)	(0.027)	**(0.027)**	(0.027)
Urinary tract infections	0.662	0.656	0.695	0.693	0.698	0.695	0.681	0.681	**0.712**	0.705
	(0.015)	(0.015)	(0.016)	(0.015)	(0.014)	(0.015)	(0.019)	(0.019)	**(0.014)**	(0.015)

**Fig. 4:**
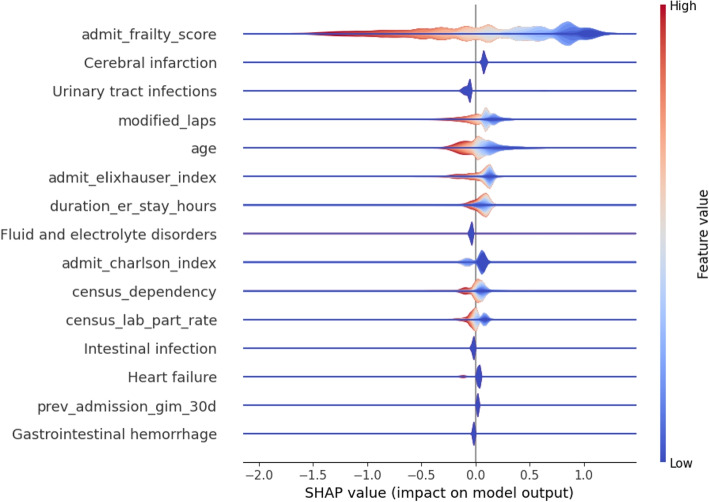
Model explanation via Shapley value to present feature attributions of top 15 features

To understand which feature contributed more to prediction, in Fig. 4, we plotted the summary of the Shapley values for all patients of the one-size-fits-all XGBoost enhanced Cox model. Figure 4 displays the top 15 features, ranked by their contribution to the prediction result. Among these 15 features: i) Six are disease diagnosis indicators: “cerebral infarction”, “urinary tract infection”, “fluid and electrolyte disorders”, “intestinal infection”, “heart failure”, and “Gastrointestinal hemorrhage”. ii) There is also one demographic variable: “age”. iii) Two socioeconomic variables: “census_dependency” and “census_lab_part_rate” were also shown to be relevant iv) There were also four commonly used derived healthcare scores and indexes: “admit_charlson_index”, “admit_elixhauser_index”, “admit_frailty_score”, and “modified_laps”. and v) two pre-admission variables “duration_er_stay_hours” and “prev_admission_gim_30 d”. Figure 3 shows the distribution of eight non-binary features among the top 15 features.

**Fig. 5:**
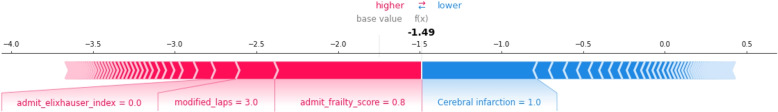
Shapley value of a certain patient

In Fig. 4, we observe that most of the top 15 selected features are related to disease type, commonly used healthcare scores/indexes, and pre-admission variables. These factors align with both established medical domain knowledge and our descriptive analysis in Fig. 2, indicating that the model can effectively identify key factors through data-driven learning. As a result, the learned knowledge is trustworthy, and the model is suitable for practical application. Moreover, as shown in Fig. 4, two socioeconomic variables play a key role in LOS prediction, demonstrating that factors like family financial burden and employment status can significantly influence the length of a patient’s hospital stay. This also suggests that addressing these socioeconomic determinants could help reduce disparities in hospital care and improve patient outcomes. Note that besides the summary plot, we can also generate a detailed plot to visualize the contribution of each feature for an individual patient, as shown in Fig. 5.

## Discussion

Leveraging survival analysis and explainable artificial intelligence (XAI) techniques for predicting in-hospital length of stay (LOS) offers numerous practical applications in healthcare, with the potential to significantly improve both patient care and clinical operations. Below are some practical applications:

1. Personalized Patient Care and Early Intervention: Combining survival analysis with XAI offers valuable insights into patient characteristics and treatment factors that influence length of stay (LOS). By identifying patients at high risk for extended stays, clinicians can implement early interventions to prevent complications that may otherwise delay recovery.
2. Enhancing Clinical Research: XAI models provide transparency in prediction models by explaining the factors driving the prediction of a patient’s LOS. By leveraging XAI techniques, healthcare professionals can gain insights into the underlying data and the rationale behind model predictions. This understanding not only aids in refining clinical reasoning and decision-making but also fosters clinical research into how to reduce the impact of factors that tend to increase LOS.
3. Improving Patient Engagement and Satisfaction: By providing patients and their families with more accurate expectations about treatment outcomes and the duration of their hospital stay, hospitals can reduce uncertainty and help patients feel more informed and in control of their care. Meanwhile, this enables better planning for patient discharge, including home care, follow-up appointments, and rehabilitation, which contributes to improved post-discharge outcomes.
4. Improving Hospital Operations and Resource Allocation: By accurately forecasting patient LOS, hospitals can better manage patient flow, reducing overcrowding and avoiding delays in the admission of new patients. Specifically, accurate LOS predictions enable more effective planning in areas such as staffing, bed availability, and medical supplies.
5. Improving healthcare equity and Resource Allocation: Using survival analysis combined with explainable artificial intelligence (XAI) in predicting in-hospital length of stay (LOS) has the potential to significantly improve healthcare equity by ensuring that all patients, regardless of their background, receive timely and appropriate care. For example, using survival analysis and XAI techniques we can address questions relating to the fairness of hospital interventions, as in the following example questions. Do racialized or economically disadvantaged patients presenting with urinary tract infections experience longer hospital stays? Do women who are frail suffer more in-hospital events that delay their discharge more than frail men experience?

## Conclusion and future work

In this paper we show how survival analysis can be used to predict LOS, and how Shapley values can be used to interpret results with respect to key predictors. The methods and analytical techniques presented in this paper can be readily applied to other datasets. Future research on prediction of LOS and other healthcare outcomes amenable to survival analysis may be carried out on a wide range of data. The analyses presented in this paper could also be extended in future research in the form of predictive tools for GIM LOS that could support healthcare leaders in decision-making surrounding health equity and operational considerations such as access to care, patient flow, healthcare human resources, etc. Ideally, findings concerning risk factors for disease, and for prolonged hospital stays, would then be paired with prevention and treatment strategies that can mitigate those risks, and that can be implemented in clinical decision support systems so that they can become part of clinical workflow.

## Data Availability

The dataset used in the research is from the GEMINI program, and the database is accessible at the following link: https://geminimedicine.ca/the-gemini-database/.

## Footnotes

1 https://cran.r-project.org/web/packages/survival/index.html

2 https://xgboost.readthedocs.io/en/stable/

3 https://github.com/havakv/pycox

4 https://cran.r-project.org/web/packages/randomForestSRC/index.html

## References

[1] Lee ET, Wang J. Statistical methods for survival data analysis. Hoboken: John Wiley & Sons; 2003.

[2] Kaplan EL, Meier P. Nonparametric estimation from incomplete observations. J Am Stat Assoc. 1958;53(282):457–81.

[3] Cox DR. Regression models and life-tables. J R Stat Soc Ser B Methodol. 1972;34(2):187–202.

[4] Chen T, Guestrin C. Xgboost: A scalable tree boosting system. In: Proceedings of the 22nd acm sigkdd international conference on knowledge discovery and data mining. 2016. p. 785–794. http://dl.acm.org/doi/abs/10.1145/2939672.2939785.

[5] Ishwaran H, Kogalur UB, Blackstone EH, Lauer MS. Random survival forests. Ann Appl Stat. 2008;2(3):841–60.

[6] Katzman JL, Shaham U, Cloninger A, Bates J, Jiang T, Kluger Y. DeepSurv: personalized treatment recommender system using a Cox proportional hazards deep neural network. BMC Med Res Methodol. 2018;18:1–12. 29482517 10.1186/s12874-018-0482-1 PMC5828433

[7] Kvamme H, Borgan Ø, Scheel I. Time-to-event prediction with neural networks and Cox regression. J Mach Learn Res. 2019;20(129):1–30.

[8] Shwartz-Ziv R, Armon A. Tabular data: Deep learning is not all you need. Inf Fusion. 2022;81:84–90.

[9] Harrell FE, Califf RM, Pryor DB, Lee KL, Rosati RA. Evaluating the yield of medical tests. JAMA. 1982;247(18):2543–6. 7069920

[10] Scott M, Su-In L, et al. A unified approach to interpreting model predictions. Adv Neural Inf Process Syst. 2017;30:4765–74.

[11] Cox DR. Partial likelihood. Biometrika. 1975;62(2):269–76.

[12] Nelson W. Hazard plotting for incomplete failure data. J Qual Technol. 1969;1(1):27–52.

[13] Auditor General of Ontario. 2010 Annual Report. 2010. Chapter 3: Reports on Value-for-money Audits. Section 3.02: Discharge of Hospital Patients. https://www.auditor.on.ca/en/content/annualreports/arreports/en10/302en10.pdf. Accessed 2 Mar 2025.

[14] Canadian Institute for Health Information. Hospital stays in Canada, 2021–2022. https://www.cihi.ca/en/hospital-stays-in-canada-2021-2022. Accessed 2 Mar 2025.

[15] Statistics Canada. 2021 Census of Population. 2022. Catalogue number: 98-10-0001. https://www.statcan.gc.ca. Accessed 15 Oct 2024.

[16] Gilbert T, Neuburger J, Kraindler J, Keeble E, Smith P, Ariti C, et al. Development and validation of a Hospital Frailty Risk Score focusing on older people in acute care settings using electronic hospital records: an observational study. Lancet. 2018;391(10132):1775–82. 29706364 10.1016/S0140-6736(18)30668-8 PMC5946808

[17] Escobar GJ, Greene JD, Scheirer P, Gardner MN, Draper D, Kipnis P. Risk-adjusting hospital inpatient mortality using automated inpatient, outpatient, and laboratory databases. Med Care. 2008;46(3):232–39. 10.1097/MLR.0b013e3181589bb6 18388836

[18] Elixhauser A, Steiner C, Harris DR, Coffey RM. Comorbidity measures for use with administrative data. Med Care. 1998;36(1):8–27. 10.1097/00005650-199801000-00004 9431328

[19] Charlson ME, Pompei P, Ales KL, MacKenzie CR. A new method of classifying prognostic comorbidity in longitudinal studies: development and validation. J Cronic Dis. 1987;40(5):373–83. 10.1016/0021-9681(87)90171-8 3558716

[20] Lee C, Zame W, Yoon J, Van Der Schaar M. Deephit: a deep learning approach to survival analysis with competing risks. Proceedings of the AAAI Conference on Artificial Intelligence. 2018;32(1). 10.1609/aaai.v32i1.11842.

[21] Yu CN, Greiner R, Lin HC, Baracos V. Learning patient-specific cancer survival distributions as a sequence of dependent regressors. Adv Neural Inf Process Syst. 2011;24:1-9.

[22] Li Y, Wang J, Ye J, Reddy CK. A multi-task learning formulation for survival analysis. In: Proceedings of the 22nd ACM SIGKDD international conference on knowledge discovery and data mining, 2016. p. 1715–1724. https://dl.acm.org/doi/abs/10.1145/2939672.2939857.

[23] Therneau TM. A package for survival analysis in R. 2024. R package version 3.6-4. https://CRAN.R-project.org/. Accessed 23 Oct 2024.

